# Time to immunologic recovery and determinant factors among adults who initiated ART in Felege Hiwot Referral Hospital, northwest Ethiopia

**DOI:** 10.1186/s13104-017-2602-0

**Published:** 2017-07-14

**Authors:** Lemma Derseh Gezie, Kassahun Alemu Gelaye, Abebaw Gebeyehu Worku, Tadesse Awoke Ayele, Destaw Fetene Teshome

**Affiliations:** 1 Department of Epidemiology and Biostatistics, Institute of Public Health, College of Medicine and Health Sciences, University of Gondar, Gondar, Ethiopia; 2 Department of Reproductive Health, Institute of Public Health, College of Medicine and Health Sciences, University of Gondar, Gondar, Ethiopia

**Keywords:** Time to immunologic recovery, CD4 count, Frailty, Ethiopia

## Abstract

**Background:**

CD4 cells are the major targets for human immunodeficiency virus (HIV) and treatment with Antiretroviral Therapy (ART) influences the CD4 cell count of HIV patients. In addition to ART, the time required to reach normal range of CD4 counts (500 cells/mm^3^) can be affected by clinical, socio-demographic, and behavioral factors. This retrospective cohort study was conducted to determine the incidence of having the normal range of CD4 cell counts and factors that affect the time required to reach this normal range among adult HIV patients who initiated into ART.

**Methods:**

Data of 4 years were retrospectively retrieved from routinely registered characteristics of 937 ART users enrolled in 2010. Survival time until immunologic recovery and its determinant factors were examined using the frailty model with different parametric distributions alternatively.

**Results:**

Most (80.8%) of the ART attendants had CD4 cell count of 200 cells/mm^3^ or less at initiation. The overall incidence rate of immunologic recovery was 12.67 persons per 1000 person-months (95% CI 11.30, 14.20). The dependency of frailties of immunologic recovery by residence was statistically significant (Theta = 0.05, p value = 0.006). Baseline age (Adjusted Hazard Ratio (AHR) = 0.98, 95% CI 0.97, 0.99), baseline CD4 count (AHR = 1.006, 95% CI 1.005, 1.008), and female sex (AHR = 1.34, 95% CI 1.03, 1.73) were significantly associated with shorter survival time for immunologic recovery.

**Conclusion:**

Higher baseline CD4 count, lower baseline age, and female sex were positively associated with the time to immunologic recovery, which also dependent on proximity/residence of ART users. Therefore, further scale up of ART services with due emphasis to patients with low CD4 count at baseline particularly for male and older ART users are recommended to reach the normal range of CD4 count in a shorter time of treatment.

## Background

Human immunodeficiency virus (HIV) remains a public health problem in Ethiopia with an estimated national prevalence of 1.14% in 2014 [1]. Out of these patients, 367,000 were taking Antiretroviral Therapy (ART); currently free ART service is available at 1045 health facilities in the country [2]. One of these health facilities is Felege Hiwot Referral Hospital which has served a large number of patients living in northwestern part of the country. For instance, currently about 5575 HIV patients are getting ART services in this hospital; 5391 of these are adults.

The ART drug changes a fatal disease to a manageable chronic illness, and a successful use of ART suppresses HIV viral replication, consequently slowing down disease progression, improving immunity, and delaying mortality [3]. In general, it has been recommended that it will be more effective when initiated as early as possible, specifically the World Health Organization (WHO) in 2015 recommended that adults should initiate ART immediately after confirming they are positive for the virus irrespective of their CD4+ T-cell (CD4) count [4].

Nevertheless, because of the high cost of medications and treatment, ART drugs are not initiated at the time of confirmation of the virus in the blood, especially for people in developing countries. For instance, Ethiopia set a cutoff point of CD4 cell count of initiating ART for adults at 200 cells/mm^3^ in 2003 and 500 cells/mm^3^ in 2014 [2]. It is in the course of time of using the drug that their CD4 counts reach, for example, the normal range of CD4 counts for the general adult population which has a minimum value of 500 cells/mm^3^ and in this study we defined it as immunologic recovery [5]. One of the aims of the treatment is to shorten this time and to help patients retain their former better immunity level as early as possible which is unclear whether this is happening in the current study area or not.

However, there are a variety of factors other than HIV that could influence the CD4 cell count of human beings. For instance, older people have poorer response to ART treatment [6, 7]. In addition inaccessibility or distance of health facility from residence [8] which can lead to late presentation that could affect the disease progression [9, 10] are the other factors that negatively influence CD4 count during treatment. Similarly, female sex was negatively associated with CD4 count [10–12] although no such association was noted in other studies [6, 13].

For a proper management of treatment, it is vital to measure the average length of time from the initiation of ART to the time until which the normal range of CD4 count is retained. It is also important to measure how different variables affect survival time. Therefore, the incidence density of immunologic recovery and the effect of socio-demographic and clinical variables on the time to immunologic recovery were determined after controlling for other variables which are not recorded in the registration books of adult ART attendants at Felege Hiwot Referral Hospital, northwest Ethiopia, by using statistical techniques of frailties of immunologic recovery among patients.

## Methods

### Study area and period

The study was conducted at Felege Hiwot Referral Hospital located in Bahir Dar, the capital of the Amhara Regional State, Ethiopia. The hospital provides ART services to eligible HIV patients in a clinic for both self and physician referred patients. The study used data of ART attendants enrolled in the first 6 months of the year 2010 and followed up to mid 2014.

### Study design

A retrospective cohort study was conducted to determine the incidence of having normal range of CD4 cell count for the general adult population and factors that influence the length of time required to reach this normal range among adult HIV patients who initiated ART in the hospital.

### Study population

All adult HIV positive persons who initiated ART treatment in the hospital from January to June, 2010 (half a year) were included in the sample. Therefore, a total of 937 patients were selected for the sample for analysis. During data collection, children aged below 18 years and pregnant women were excluded and efforts were made to maintain the quality of the data.

### Data collection procedures

A pretested structured data collection checklist was used to extract routinely recorded data from adult HIV patients who initiated ART treatment in the hospital. All charts containing detailed data, including the baseline CD4 count and repeated measures of CD4 cell counts almost every 6 month were reviewed. Similarly, other characteristics, like socio-demographic (age and sex), clinical (baseline WHO clinical stage, baseline functional status), residence, baseline regimen together with others were also collected from the registration book of ART users.

Five health professionals (B.Sc. Nurses) trained for 2 days retrieved the data. The principal investigator and the supervisor closely monitored the process throughout the data collection period and made due corrections.

Survival time was defined as the time starting from the date of initiation of ART to the time it reaches the minimum value of the normal range of CD4 count (500 cells/mm^3^) determined for each participant. Participants who never attained the normal range for different reasons over the follow up period were considered as right censored.

### Data processing and analysis

The edited and cleaned data were analyzed using the statistical software called STATA version 12. The data were processed to generate the survival time to reach the normal range of CD4 count and the censoring indicator. Texts, tables, and graphs were used to show frequencies of socio-demographic, clinical, and other variables. The survival time until patients’ CD4 count reached normal range was also described. Incidence rate of immunologic recovery by each characteristic of patients, including an overall incidence rate were determined.

As the study was based on secondary data, all relevant independent variables that could affect the survival time might not be available. Controlling the effect of these unmeasured variables is important to minimize bias on the estimate of the effect of existing variables. Therefore, the frailty model was employed to determine the adjusted effect of available variables on the event of interest after controlling for variables not included in the model. The effect of unmeasured variables on survival time were shared into the residence of participants which was categorized as remote and close residences, and the statistical method used was the shared frailty model.

Before using the survival regression models, the assumption of proportional hazard was checked using the log–log technique. Weibull, exponential, and lognormal distributions were used alternatively to get the fit of the baseline hazard. The gamma and inverse Gaussian distributions were also used alternatively to represent the distribution of frailty. For nested models the likelihood ratio test was used, while for non-nested models the Akaike Information Criteria was employed to see the fit of data. A p value of 0.05 or less was considered to be statistically significant.

## Results

### Patient characteristics

A total of 937 HIV positive adults who initiated ART treatment at Felege Hiwot Referral Hospital were included in the study. The average age of the participants was 33.7 (95% CI 33.1, 34.3) years; 530 (56.6%) of whom were females. Of the participants, 585 (62.4%), lived in Bahir Dar (location of hospital) and in neighboring towns.

Out of the whole participants, 655 (69.9%) had Baseline WHO Clinical Stage III and 642 (68.5%) had Working Functional Status. Including the baseline CD4 count measurement, the minimum and maximum numbers of CD4 count readings taken from each study participant were 1 and 9, respectively. The most important reasons for the incompleteness of data or right censoring were death, transfer out to other health facilities, and loss to follow up (Table 1).

**Table 1:** Characteristics of ART attendants, Felege Hiwot Referral Hospital, 2010–2014

Characteristics	Number (%)
Baseline age in year (n = 937)	
18–25	173 (18.5)
26–30	267 (28.5)
31–40	332 (35.5)
41–50	114 (12.2)
51–71	51 (5.4)
Sex (n = 937)	
Female	530 (56.6)
Male	407 (43.4)
Residence	
Bahir Dar and neighboring towns	585 (62.4)
Others	352 (37.6)
Baseline functional status (n = 937)	
Working	642 (68.5)
Ambulatory	246 (26.3)
Bedridden	49 (5.2)
Baseline WHO clinical stage (n = 937)	
Stage I	44 (4.7)
Stage II	133 (14.2)
Stage III	655 (69.9)
Stage IV	105 (11.2)
First treatment class (n = 937)	
AZT-3TC	553 (59.0)
D4t-3TC	29 (3.1)
D4t (30)	355 (37.9)
Baseline CD4 count in cells/mm^3^ (n = 937)	
<200	757 (80.8)
200–349	172 (18.3)
350–499	8 (0.9)

### CD4 cell counts

The participants were followed for a total of 23,136 person-months, and 289 (30.8%) participants reached the normal range of CD4 count for the general adult population (500 cells/mm^3^ or more) during the follow up period. The overall incidence rate of reaching the normal range of CD4 count was 12.67 persons per 1000 person-months (95% CI 11.3–14.2). The incidence rate of reaching the normal range of CD4 counts among age groups of 18–25 years was 17.20 persons per 1000 person-months (95% CI 13.6, 21.9) while it was 4.19 persons per 1000 person-months (95% CI 1.8, 9.1) for the age groups of 51–71 years. The incidence rate of reaching the normal range for individuals from Bahir Dar where the hospital is located was about 15.05 persons per 1000 person-months, while for those who came from remote districts it was only 7.21 persons per 1000 person-months. The incidence rate of reaching the normal range of CD4 counts for patients who had baseline CD4 counts of lower than 200 cells/mm^3^ were 9.70 persons per 1000 person-months (95% CI 8.4, 11.1), while it was 44.4 persons per person-months (95% CI 16.7, 118.5) for patients with baseline CD4 counts of 350–499 cells/mm^3^ (Table 2).

**Table 2:** Number of events of reaching normal range of CD4 count and incidence densities by patient characteristics among adult HIV patients who initiated ART in Felege Hiwot Referral Hospital, 2010–2014

Characteristics	Person-months	Number of events	Incidence density per 1000 person-months (95% CI)
Baseline age in year (n = 937)			
18–25	3888	67	17.2 (13.6, 21.9)
26–30	6174	76	12.3 (9.8, 15.4)
31–40	8610	111	12.9 (10.7, 15.5)
41–50	3042	29	9.5 (6.6, 13.7)
51–71	1470	6	4.2 (1.8, 9.1)
Sex (n = 937)			
Female	12,630	195	15.5 (13.5, 17.8)
Male	10,554	94	8.9 (7.3, 10.9)
Residence			
Bahir Dar and neighboring towns	15,552	234	15.1 (13.2, 17.1)
Others	7632	55	7.2 (5.5, 9.4)
Baseline functional status (n = 937)			
Working	17,466	220	12.6 (11.0, 14.4)
Ambulatory	4830	61	12.6 (9.8, 16.2)
Bedridden	888	8	9.0 (4.5, 18.0)
Baseline WHO clinical stage (937)			
Stage I	1200	13	10.8 (6.3, 18.7)
Stage II	3768	35	9.3 (6.7, 12.9)
Stage III	1598	211	13.2 (11.5, 15.1)
Stage IV	2232	30	13.4 (9.4, 19.2)
First treatment class (n = 1196)			
AZT-3TC	14,514	166	11.4 (9.8, 13.3)
D4t-3TC	804	11	13.7 (7.6, 24.7)
D4t (30)	7866	112	14.2 (11.8,17.1)
Baseline CD4 count in cells/mm^3^ (n = 1196)			
<200	19,848	192	9.7 (8.4, 11.1)
200–349	3246	93	28.7 (23.3, 35.1)
350–499	23,184	4	44.4 (16.7, 118.5)

**Fig. 1:**
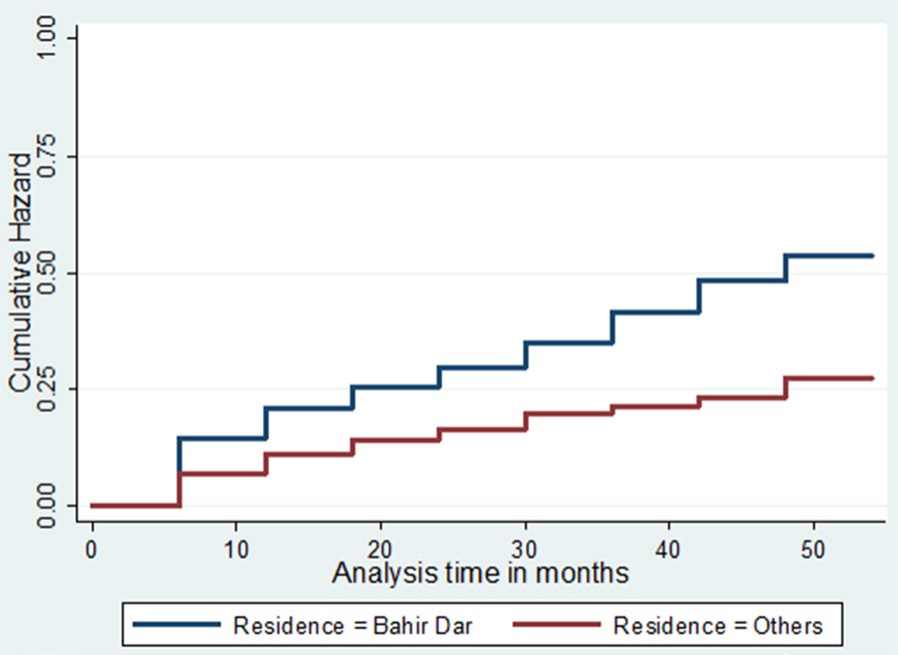
Cumulative hazard of reaching the normal range of CD4 count among ART users in Felege Hiwot Referral Hospital, northwest Ethiopia, 2014

The cumulative hazard of reaching the normal range of CD4 count was higher for females than males (p value <0.001). Similarly, participants living in Bahir Dar had a higher cumulative hazard of reaching the normal range of CD4 count compared to others (p value <0.001) (Fig. 1).

### Determinant factors for time to reaching the normal range of CD4 count

Before trying to fit the data with any of the models, the proportional hazard assumption was checked for each of the covariates using the log–log technique, and the results were satisfactory to assume the lines were more or less parallel. Therefore, six variables including participants’ baseline age, sex, baseline CD4 count, baseline functional status, baseline WHO clinical stage, and the type of first line drug were considered as independent variables.

To account for the effect of unmeasured variables, the frailty model was fitted. To meet this aim, the effect of unmeasured variables was analyzed by sharing it to the categories of residence, Bahir Dar and neighboring towns where the hospital is situated on one hand and other districts in the catchment area on the other hand. Here, residence was preferred on the assumption that it could accommodate the effect of different characteristics, like accessibility, information, adherence, etc. as individuals in the second group were mostly from remote rural areas.

Different techniques of survival analysis were tried to find a model that best fits the data. First, the proportional hazard model was fitted; then, the Weibull, exponential, and log-normal parametric survival models were fitted. Finally, with these parametric models, the gamma and inverse Gaussian univariate shared frailty models out of which the one that gave the best fit was selected were fitted.

Accordingly, a model with a baseline hazard of Weibull distribution with a shared frailty of gamma distribution was selected as the best fit to the data. Thus, three variables including age, sex, and baseline CD4 count were found to be statistically significantly associated with the hazard of getting into the normal range of CD4 counts. For instance, for each additional age in years, the hazard of reaching the normal range of CD4 count decreased by 2% (AHR = 0.980, 95% CI 0.966, 0.994) (Table 3).

**Table 3:** Determinant factors of time to reach the normal range of CD4 count among adult HIV patients who initiated ART in Felege Hiwot Referral Hospital, 2010–2014

Characteristics	Hazard ratio (95% CI)
Baseline age in years	0.980 (0.966, 0.9941)
Sex	
Female	1.0
Male	0.747 (0.577, 0.968)
Baseline functional status	
Working	1.0
Ambulatory	1.005 (0.742, 1.362)
Bedridden	0.910 (0.441, 1.879)
Baseline WHO clinical stage	
Stage I	1.0
Stage II	1.128 (0.593, 2.146)
Stage III	1.567 (0.887, 2.769)
Stage IV	1.514 (0.776, 2.952)
First treatment class	
AZT-3TC	1.0
D4t-3TC	1.223 (0.950, 1.575)
D4t (30)	1.243 (0.672, 2.298)
Baseline CD4 count	1.006 (1.005, 1.008)
P	1.186 (1.082, 1.299)
Theta	0.052 (0.005, 0.551)

P is the shape parameter of Weibull distribution; Theta is the parameter for the frailty

The shape parameter, P, was estimated to be 1.186 (95% CI 1.082, 1.299). The dependency of the variability of frailties on the categories of residence was also captured by the parameter ‘theta’ and was estimated to be 0.0523 (p value = 0.006).

## Discussions

The current study included 937 adult ART users who initiated the treatment during the first half year of 2010 and were followed retrospectively up to mid 2014. For the majority of the participants (757 or 80.8%), the baseline CD4 count was less than 200 cells/mm^3^ as it was one of the criteria to enroll for ART for HIV positive persons, and the remaining persons (180 or 19.2%) could have enrolled by other criteria, mostly considering their WHO clinical stage. Baseline CD4 count, baseline age, and sex were estimated to be determinants of time to immunologic recovery.

The baseline hazard (the effect of time when all categorical variables are at a reference category and when all continuous covariates are at zero) of reaching the normal range of CD4 count was fitted with Weibull distribution with a shape parameter (P) greater than one (P = 1.186; 95% CI 1.082, 1.299). The Weibull shape parameter greater than one implies that the hazard of immunologic recovery increases with time of treatment.

In the analysis of frailty model, residence was taken as a clustering variable. The variability of the effect of unobserved variables on the time to immunologic recovery was estimated to be significantly different from zero (Theta = 0.052, p value = 0.006). This shows that there are unmeasured confounders which have a significant effect on the time to the event of interest, and the distribution of these unmeasured variables is different between ART users from Bahir Dar city and those who come from districts. This could be so probably because in addition to being one of the big cities in the country, Bahir Dar is a place where the hospital that ART users get treatment is located. There could be a suitable condition for patients under treatment so that the time required for immunologic recovery could be short for patients who are residing in the city than others who came from the surrounding mostly from rural districts. Different underlying and unmeasured characteristics that could affect adherence to ART, like accessibility [14–16], information, and other societal characteristics that could probably affect the time to immunologic recovery could be different across such residences.

Baseline CD4 count was positively associated with the time to the event of immunologic recovery or reaching the normal range of CD4 count for the general adult population (500 cells/mm^3^). With different statistical methods, other studies also reached the same conclusion [17, 18]. This could be of course justifiable because for those who had a higher baseline CD4 count, it will be easier to reach the normal range as they are closer to the range than others who had far a lesser CD4 count. Patients with smaller CD4 counts could have a suppressed immunity [16] that could make retaining the normal range of CD4 count difficult for them or they could have co-infections which could make the immunologic recovery difficult [19].

Baseline age was negatively associated with the time required to reach the normal range of CD4 count. Other different studies supported this finding [7, 18]. Different authors reasoned out that a favorable influence of younger age on CD4 cell recovery while a patient is receiving ART may be explained by more-effective thymic function [18]. This is because of different biological characteristics, including immunity that changes with age. It was also shown that older persons come to the clinic late [15] that could make their response to ART treatment poorer [16]. Contrary to these findings, other studies reported a null association between age and CD4 count increment [13, 19]. This could probably be so because the majority of the patients were aged less than 50 years [13], and 50% of the participants were between 31 and 41 years of age [19] in the respective studies, and in both cases the relative homogeneity in age could dilute its effect on CD4 count increment.

In this study, sex was an independent predictor of the hazard of reaching CD4 count, specifically, males reduce the hazard of reaching normal range of CD4 count almost by 25% when compared to females (AHR = 0.747, 95% CI 0.577, 0.968). Other studies were consistent with this finding [5, 10]. Contrary to these studies, other authors reported that there was no significant difference between males and females in response to ART [16, 18]. There could be different reasons why a response to ART between males and females were different among the studies. One justification is that females could attend Voluntary Counseling and Testing as part of their routine health care services during pregnancy. As a result, they are more likely to be diagnosed for infections earlier than males [20], and this could make the response to ART treatment poorer for late diagnosed males as their immunity could be weakened. However, early presentation and other related factors might be indifferent for males and females in the studies which reported insignificant associations.

In this study, baseline functional status was not an independent predictor of the time to immunologic recovery. However, a study that used longitudinal data analysis showed statistically significant association between baseline functional status and CD4 count [21]. Probably, this difference could be the distinction in the variables predicted by the two studies. The current study predicted the time to immunologic recovery while the study done in eastern Ethiopia predicted the CD4 count. Therefore, functional status may not have any effect on the time to reaching the normal range of CD4 count.

As a limitation, because the study was based on secondary data, information related with some relevant variables (e.g. nutritional status, substance use, etc.) was not available; hence, their effect on the time to immunologic recovery was not determined. However, the frailty model was used to determine the effect of observed variables after controlling for the unmeasured ones.

## Conclusion

Higher baseline CD4 count, lower baseline age, and female sex were positively associated with time to immunologic recovery of CD4 count. It was also dependent on residence of ART users. Therefore, further scale up of ART services to be accessible to their residence with due emphasis to patients with low CD4 count at baseline particularly for male and older ART users are recommended to reach normal range of CD4 count in shorter time of treatment.

## Abbreviations

AHR: Adjusted Hazard Ratio
ART: Antiretroviral Therapy
CD4: cluster of differentiation 4
HIV: human immune virus
WHO: World Health Organization

## References

[1] WHO. Ethiopia|HIV/AIDS. Regional office for Africa. 2015.

[2] Frehiwot N, Mizan K, Seble M (2014). National guidelines for comprehensive HIV prevention, care and treatment.

[3] Hammer SM, Squires KE, Hughes MD (1997). A controlled trial of two nucleoside analogues plus indinavir in persons human immunodeficiency virus infection and CD4 cell counts of 200 per cubic millimeter or less. AIDS Clinical Trails Group 320 Study Team. N Engl J Med.

[4] World Health Organization. Guideline on when to start ART and on pre-exposure prophylaxis for HIV. 2015. 26598776

[5] Kaufmann RG, Perrin L, Pantaleo G (2003). CD4 T-lymphocyte recovery in individuals with advanced HIV-1 infection receiving potent antiretroviral therapy for 4 years: the Swiss HIV Cohort Study. Arch Intern Med.

[6] Montarroyos RU, Miranda-Filho CD, César CC (2014). Factors related to changes in CD4+ T-cell counts over time in patients living with HIV/AIDS: a multilevel analysis. PLoS ONE.

[7] Florence E, Lundgren J, Dreezen C (2003). Factors associated with a reduced CD4+ lymphocyte count response to HAART despite full viral suppression in the EuroSIDA study. HIV Med.

[8] Bonjour MA, Montagne M, Zambrano M (2008). Determinants of late disease-stage presentation at diagnosis of HIV infection in Venezuela: a case–case comparison. AIDS Res Ther.

[9] Louis C, Ivers LC, Smith FM, Freedberg KA, Castro A (2007). Late presentation for HIV care in central Haiti: factors limiting access to care. AIDS Care.

[10] Shastri S, Boregowda HP, Rewari BB, Tanwar S, Shet A, Kumar AMV (2013). Scaling up antiretroviral treatment services in Karnataka, India: impact on CD4 counts of HIV-infected people. PLoS ONE.

[11] Malaza A, Mossong J, Barnighausen T, Viljoen J, Newell ML (2013). Population-based CD4 counts in a rural area in South Africa with high HIV prevalence and high antiretroviral treatment coverage. PLoS ONE.

[12] Addisu A, Dagim A, Tadele E, Filmon K (2015). CD4 cell count trends after commencement of antiretroviral therapy among HIV infected patients in Tigray, Northern Ethiopia: a retrospective cross-sectional study. PLoS ONE.

[13] Smith JC, Sabin AC, Youle SM (2004). Factors influencing increases in CD4 cell counts of HIV positive persons receiving long-term highly active antiretroviral therapy. J Infect Dis.

[14] Lankowski JA, Siedner JM, Bangsberg RD, Tsai CA (2009). Count restoration in HIV-infected patients receiving long-term antiretroviral treatment. Clin Infect Dis.

[15] Kwobah CM, Braitstein P, Koech JK (2015). Factors associated with late engagement to HIV care in Western Kenya: a cross-sectional study. J Int Assoc Provid AIDS Care..

[16] Gea-Banacloche J, Clifford LH (1999). Immune reconstitution in HIV infection. AIDS.

[17] Paddam AL, Weber NJ, Frater JA, Dunn TD, Pocock JS. Effect of baseline CD4 cell count on immunological response to antiretroviral therapy in therapy-naive HIV-1—infected individuals: analysis of the St. Mary’s clinical database [abstract TuPeB4662]. In: Program and abstracts of the XIV International AIDS Conference (Barcelona). Stockholm: International AIDS Society. 2002;1:430.

[18] Viard PJ, Mocroft A, Chiesi A (2001). Influence of age on CD4 cell recovery in human immunodeficiency virus-infected patients receiving highly active antiretroviral therapy: evidence from the EuroSIDA study. J Infect Dis.

[19] Kim K-H, Yi J, Lee HS (2015). The CD4 slope can be a predictor of immunologic recovery in advanced HIV patients: a case–control study. J Intern Med.

[20] Mojumdar K, Vajpayee M, Chauhan NK, Mendiratta S (2010). Late presenters to HIV care and treatment, identification of associated risk factors in HIV-1 infected Indian population. BMC Public Health.

[21] Reda AA, Biadgilign S, Deribew A, Gebre B, Deribe K (2013). Predictors of change in CD4 lymphocyte count and weight among HIV infected patients on anti-retroviral treatment in Ethiopia: a retrospective longitudinal study. PLoS ONE.

